# Occurrence and Characteristics of Extended-Spectrum β-Lactamase-Producing *Escherichia coli* from Dairy Cattle, Milk, and Farm Environments in Peninsular Malaysia

**DOI:** 10.3390/pathogens9121007

**Published:** 2020-11-30

**Authors:** Emelia Aini Kamaruzzaman, Saleha Abdul Aziz, Asinamai Athliamai Bitrus, Zunita Zakaria, Latiffah Hassan

**Affiliations:** 1 Department of Veterinary Pathology and Microbiology, Faculty of Veterinary Medicine, Universiti Putra Malaysia, Serdang 43400 UPM, Selangor, Malaysia; emelia@dvs.gov.my (E.A.K.); bitrusaa@unijos.edu.ng (A.A.B.); zunita@upm.edu.my (Z.Z.); 2 Department of Veterinary Microbiology and Pathology, Faculty of Veterinary Medicine, University of Jos, Jos PMB 2084, Plateau, Nigeria; 3 Institute of Bioscience, Universiti Putra Malaysia, Serdang 43400 UPM, Selangor, Malaysia; 4 Department of Veterinary Laboratory Diagnostics, Faculty of Veterinary Medicine, Universiti Putra Malaysia, Serdang 43400 UPM, Selangor, Malaysia; latiffah@upm.edu.my

**Keywords:** ESBL *Escherichia coli*, dairy cattle, milk, ESBL genes, farm environment

## Abstract

The emergence and spread of antimicrobial resistance genes and resistant bacteria do not recognize animal, human, or geographic boundaries. Addressing this threat requires a multidisciplinary approach involving human, animal, and environmental health (One Health) sectors. This is because antimicrobial agents used in veterinary medicine have been reported to be the same or similar to those in human medicine use. Extended-spectrum β-lactamase (ESBL) *E. coli* is a growing public health problem worldwide, and the agri-food industry is increasingly becoming a source of clinically important ESBL bacteria. Accordingly, the aim of this study was to investigate the occurrence and characteristics of ESBL-producing *E. coli* from dairy cattle, milk, and the farm environment. *E. coli* isolates were identified by their 16sRNA, and their ESBL production was confirmed using ESBL–CHROMagar media and the double disk diffusion method. Genotypes of ESBL producers were characterized using multiplex polymerase chain reaction (mPCR) assay. It was found that 18 (4.8%) of the total samples were positive for ESBL-producing *E. coli*. Of these, 66.7% were from milk, and 27.8% and 5.5% were from the farm environment and faecal samples, respectively. Predominant ESBL genotypes identified were a combination of both TEM and CTX-M in eight out of 18 (44.4%) isolates. Four (22.2%) isolates produced the CTX-M gene, two (11.1%) isolates produced the TEM gene, and four (22.2%) remaining isolates produced the ESBL genes other than TEM, SHV, CTX-M, and OXA. The SHV and OXA gene were not detected in all 18 isolates. In all, there were four profiles of genetic similarity. The occurrence of these genotypes in indicator organisms from dairy cattle, milk, and the farm environment further re-enforced the potential of food-animals as sources of ESBL-producing *E. coli* infection in humans via the food chain. Thus, there is the need for the adoption of a tripartite One Health approach in surveillance and monitoring to control antimicrobial resistance.

## 1. Introduction

The remote cause and actual cost of antimicrobial resistance (AMR) in most parts of the world remains unclear despite being a significant public health problem. As a complex global health problem, tackling the menace of antimicrobial resistant bacteria requires a holistic transdisciplinary approach involving human, animal, and environmental health sectors, collectively tagged the One Health approach. The concept of One Health to control AMR in bacteria through active surveillance and monitoring has been recognized as a priority action that can facilitate better understanding of AMR. AMR surveillance program in the agri-food sector considers commensal bacteria such as *E. coli* as an indicator organism because AMR profiles in *E. coli* almost accurately mirrors the use of antimicrobial agents in food-animals [1,2], which can transfer and spread to pathogenic bacteria.

*Escherichia coli* producing narrow and extended spectrum β-lactamases (ESBL) are continuously becoming a public health problem worldwide [3,4]. ESBL are plasmid-mediated β-lactamase enzyme recognized for their remarkable ability to hydrolyse penicillin, 3rd and 4th generation cephalosporins and monobactams except for carbapenem and cephamycin [5]. These enzymes emerged from *bla*TEM-1, *bla*TEM-2, and blaSHV as narrow-spectrum parents. Recently, blaCTX-M, a new class of ESBL genes, appeared to have gained global traction, because of the burden it placed on animal and human health. Amino acid sequence analysis of CTX-M variants grouped these enzymes into five distinct clusters including CTX-M-1, CTX-M-2, CTX-M-8, CTX-M-9, and CTX-M-25 [4,5]. The successes of these genes in the ecosystem may likely be associated with the spread of bacterial strains carrying ESBL genes and horizontal transfer of these genes on transmissible plasmids [6,7,8]. Thus, making identification of sources and routes of transmission of ESBL-producing *E. coli* difficult. The mechanism of resistance to β-lactams in *E. coli* is majorly based on the inactivation of the β-lactams antibiotics via hydrolysis of their β-lactam rings that are catalysed by β-lactamase enzymes. *E. coli* isolates that carry ESBL genes can hydrolyse almost all cephalosporins and penicillin [8,9,10]. ESBL enzymes are mostly found in *Enterobacteriaceae* and often exhibit multi-drug resistance against non-β-lactams antimicrobial agents [4,11].

Antimicrobial resistance (AMR) is a growing problem in veterinary medicine, because it involves various species of animals and microorganisms, different breeding and animal rearing environment as well as resistance mechanisms [9,12]. Farm animals including poultry, pigs, beef, and dairy cattle have been reported as potential sources of antimicrobial resistant bacteria and resistance genes [7,13]. These resistant bacteria may transfer resistance genes horizontally to other bacteria and may pose a risk to public health via the food chain. However, resistant bacteria are not only limited to food-animals. Food-animal-derived products—namely, meat, milk, and cheese—have been reported as potential sources of resistant zoonotic bacteria [4,11,14]. Farm environments including soils, water, pests, and workers have also been reported to carry these harmful pathogens, which contribute to the dissemination and maintenance of resistance genes in the environment [10,15,16]. This situation may cause serious bilateral implications to farm animals and human. Food-producing animals have been recognized to act as carriers of several zoonotic pathogens including beta lactamase-producing *E. coli* [17]. Hence, this study aimed to investigate the occurrence and characteristics of ESBL-producing *E. coli* from dairy cattle, milk, and farm environments in the states of Selangor and Negri Sembilan, Malaysia.

## 2. Results

**Figure 1:**
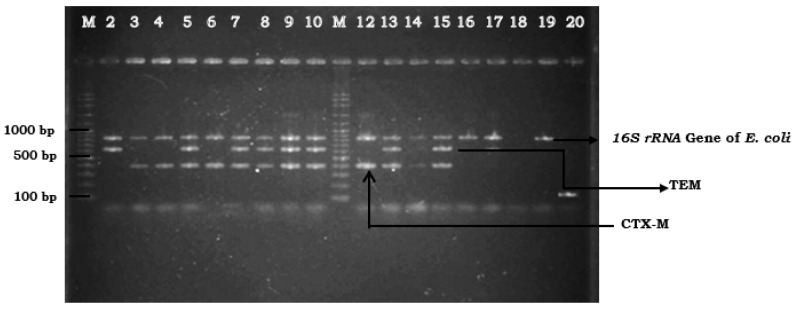
Extended spectrum beta lactamase (ESBL) genes detected using multiplex PCR assay: Lanes M: marker 100 bp ladder; Lanes 2 to 10, and Lanes 12 to 17: ESBL-producing *E. coli* isolates; Lane 18: negative control; Lane 19: *E. coli* ATCC 25922; Lane 20: *K. pneumonia* ATCC 700603.

Out of the 377 samples collected and examined for ESBL production, 18 (4.8%) were positive for ESBL *E. coli*. Of these, only one cattle (0.4%) from a total of 229 faecal samples was positive for ESBL-producing *E. coli*, and seven farms had either faeces, environment, and/or milk samples positive. ESBL-producing *E. coli* were not detected in faeces, environment, or milk samples in three farms (farm 6, farm 8, and farm 10). Among all farms, the highest occurrence of ESBL-producing *E. coli* was 15.9% in farm 4. The highest occurrence of ESBL-producing *E. coli* in milk samples was also from farm 4, where six of seven (8.5%) isolates were detected (Table 1). For the farm environment, ESBL-producing *E. coli* were detected in drinking water at 3/77 (3.9%), and one isolate (1.3%) each from water source and house flies, respectively (Table 2). ESBL-producing *E. coli* was not detected from floor, feed, and water trough swabs as well as feed samples. Genotypic detection of ESBL genes produced by *E. coli* isolates identified in this study was dominated by gene combination of both TEM and CTX-M in eight of 18 (44.4%) isolates. Four (22.2%) isolates produced CTX-M gene, two (11.1%) isolates produced TEM gene, and four (22.2%) remaining isolates produced ESBL gene other than TEM and CTX-M. The SHV and OXA gene were not detected in all 18 isolates. Thus, four genetic profiles were obtained (Table 3). Figure 1 showed genes amplified from the multiplex PCR assay.

## 3. Discussion

The emergence and spread of ESBL *E. coli* have become a public health concern, because of their association with increased morbidity and mortality, reduced treatment options, and prolonged hospital admission. The present study was designed to investigate the occurrence and characteristics of ESBL *E. coli* from dairy cattle, milk, and farm environments in Malaysia. Of the 229 faecal samples collected, only one (1) (0.4%) was positive for ESBL-producing *E. coli* isolated from a lactating cow and five (6.5%) out of 77 farm environment samples were positive. ESBL *E. coli* were mainly isolated from milk samples at 12 (16.9%) out from 71 samples. There was a statistically significant difference (χ^2^ = 32.94, *p* < 0.001) in the occurrence of ESBL-producing *E. coli* among dairy cattle, farm environments, and milk. Five (62.5%) out of eight milk samples contained ESBL *E. coli* that produced a gene combination of TEM and CTX-M. The TEM gene alone was identified from two milk samples, while the CTX-M gene alone was identified from two milk, one water source, and one drinking water sample. ESBL genotypes were not detected in four of 18 (22.2%) isolates. However, according to Schmid et al. [13], the phenotypically positive ESBL-producing *E. coli* but genotypically negative ESBL genes could also be regarded as ESBL producers because the performed m-PCRs screened for the most common resistance genes and did not include all existing resistance genes. In Malaysia, the occurrence of ESBL-producing *E. coli* in dairy cattle has not been reported prior to this study. A large percentage (>60%) on the prevalence of ESBL-producing organisms in food-animals and their products have been extensively reported by various countries particularly in the European region [13,14,15,18]. Those studies reported the occurrence of ESBL-producing organisms in food-animals including dairy cattle, poultry, and beef cattle, and in animal-based products. However, reports on ESBL-producing organisms in Asia were only limited to Japan, China, and Korea [16,19,20,21].

From this study, the occurrence of ESBL-producing *E. coli* from the selected dairy cattle farms in the states of Selangor and Negri Sembilan was considered low. The dairy industry in the country is not a major livestock industry compared to poultry and swine industries. There are many commercial-scale dairy farmers; however, most of the dairy farmers operated the farms at a small-scale level. The small-scale dairy farmers had a small herd size, which was less than 100 animals in a farm. High density of animals in a farm may provide a conducive environment for the transfer of resistant genes between animals as well as between bacterial species. Watson et al. [14] reported a high prevalence of CTX-M-15-producing *E. coli* in different cattle groups including heifers, dry cows, and high and low milk yielding groups. However, in this present study, the occurrence was found in a lactating cow (0.4%). The other dairy cattle may not be shedding *E. coli* carrying ESBL enzymes at the time of sampling, or they may be truly absent. Several other published studies reported low prevalence of ESBL-producing bacteria in cattle. In a study conducted in Japan, the prevalence of CTX-M-2 beta-lactamase among cattle was 1.5% (six of 396 cattle sampled) [14]. In another study conducted by Reist et al. [22] in Swiss cattle population younger than two years old at abattoir level, the authors reported a slightly lower (8.4%) prevalence of ESBL *E. coli*. In Korea, Tamang et al. [16] detected 0.2% ESBL-producing *E. coli* among healthy cattle. Furthermore, in Tunisia, Jouini et al. [23] found no ESBL-producing *E. coli* in cattle.

There was no association between the occurrence of ESBL-producing *E. coli* in the lactating cow group and the milk samples collected. This was also shown in a study by Geser et al. [24]. It was observed that although minced beef and pork were negative for ESBL-producing *E. coli,* the mastitic milk samples used in the study were positive. The reason could be the *E. coli* carrying ESBL genotypes in the milk may have originated from the environment. Several factors have been reported to contribute to the presence of pathogens (Shiga toxin *E. coli*, *Listeria monocytogenes*, *Salmonella*, and *Campylobacter* spp.) in milk, which included dairy farm environment hygiene, numbers of animals on the farm, farm management practices, farm workers, geographic location, and season [25].

In this study, CTX-M gene was predominantly detected in 66.7% of the isolates. This finding was consistent with the study conducted among healthy food-animals in China [20] and cattle in the Republic of Korea [16]. In a study conducted in a Malaysian hospital, it was reported that CTX-M-15-producing *E. coli* was the predominant CTX-M variant in paediatric patients [26]. However, Lim et al. [27], in their study on characterization of ESBL-producing *E. coli* isolates in a different Malaysian hospital, found a high occurrence of TEM ESBL (87.5%). Farm management and practices may have contributed to the occurrence of ESBL-producing *E. coli* in the animal and environment. Frequency of farm cleaning might also influence the low occurrence of ESBL-producing *E. coli*. The floors of the animal stalls were cleaned at least twice daily, which may help to reduce the risk of bacterial contamination to the animals and farm environment. Oliver et al. [25] reported that farm management practices contribute to the prevalence of pathogenic microorganisms in the farm. The types of animal farming whether intensive, semi-intensive, or free ranging can contribute to the development of antibiotic resistance due to the inappropriate use of antibiotics. Mixing of animal feed with antibiotics to increase feed efficiency and production levels has been a common practice in livestock management particularly in poultry and swine industries. However, it is not a common practice to mix antibiotics in dairy cattle feed. Fresh, cut, and carry grasses were given to the dairy cattle, ad. lib supplemented with dairy cattle pellet and some agricultural by-products such as molasses. Hence, such a situation may result in the low occurrence of ESBL-producing *E. coli* in this study because of less use of antibiotics at sub therapeutic level. The presence of ESBL-producing *E. coli* in raw milk may pose food safety hazards to human if milk is not heat-treated. Such resistant organisms may colonize the human intestinal tract and contribute resistance genes to human endogenous flora [28]. Timofte et al. [29] reported the first case of bovine mastitis due to ESBL-producing *E. coli* with CTX-M-15 in Europe and due to *K. pneumoniae* subsp. *pneumoniae* SHV-12 in the United Kingdom.

Nineteen percent of drinking water samples carried ESBL-producing *E. coli*. The drinking water may be contaminated with faeces of dairy cattle harbouring ESBL-producing *E. coli*. Another possible explanation was the contamination of the cattle drinking water by birds’ droppings. It was also found that flies carried ESBL-producing *E. coli,* which could spread the organisms in the environment. Surface water that comprised rivers, streams, lakes, and ponds may be the source of hazardous biological contaminants. In a study conducted in Malaysian urban surface water, Tissera and Lee [30] reported that *E. coli* and *K. pneumoniae* were predominantly isolated (89.5%), with a relatively high occurrence of CTX-M genes (84.2%), followed by TEM genes (47.4%). Similarly, Lu et al. [31] found a high diversity of ESBL-producing bacteria, with CTX-M being the most dominant gene being isolated from an urban river sediment habitat. The finding in this present study was similar, whereby CTX-M was isolated from sampled water source. Such water source if used for washing and drinking may lead to contamination of udder milking equipment and colonization in animals.

All dairy cattle farms in this study practiced open-house systems and wild birds were observed flying freely to find food and water in those farms, and hence, they may have contaminated the barn including feed and water. These birds were likely to disseminate resistance genes, as they have been reported to shed ESBL-producing *E. coli* in the environment [32]. Migratory birds have been reported to play an epidemiological role in disseminating antibiotic resistance genes and as a potential reservoir of ESBL-producing organisms [28]. Food of animal origin may play a role in disseminating ESBL-producing *E. coli* implicating mastitis in dairy cattle, which originate from the environment and were reported to occur more commonly in high producing cow at the first two weeks after calving. Cattle were most likely to get infected when they were on faeces-contaminated bedding [33]. *E. coli* may enter the teat orifice causing ascending infection of the mammary gland.

## 4. Materials and Methods

### 4.1. Study Design and Sample Collection

A total of 377 samples were collected from ten dairy cattle farms located within Selangor and Negeri Sembilan states and were examined for ESBL-producing *E. coli*. Out of 10 farms, six were small-scale, and two each were medium- and commercial-scale, respectively. The herd size of each farms also varied between 32–63 animals for small-scale, 57–157 animals for medium-scale, and 165-188 animals for commercial-scale. The samples included 229 faeces, 71 milk, and 77 farm environment samples. Each faecal sample was collected using a sterile swab and placed in 9 mL of sterile buffered peptone water (BPW) (CM0509, Oxoid, Basingstoke, Hampshire, England). Faecal samples were collected from different groups of animals, which included lactating cows (n = 69), dry cows (n = 44), heifers (n = 32), young bulls (n = 25), bulls (n = 8), and calves (n = 51). Milk samples were collected from the lactating cows from which faecal samples were taken. Approximately 20 mL of milk sample from each lactating cow were hand-milked directly into a sterile bottle. All cows were apparently healthy and did not exhibit any clinical signs of mastitis. Prior to milk sample collection, we assessed the cleanliness state and appearance of the udder for any gross contamination and aberrations. The udder was then thoroughly cleaned and wiped with a clean dry towel. Each of the teats was disinfected with 70% alcohol. Farm environment samples collected and used included swabs of stall/pen floor, house flies, and feed and water troughs. Floor swabs were collected randomly, which included the calves’ pen, areas covering dry and milking cow stall, and working bull pen. Three swabs each were collected from the floors and feed and water troughs, which were pooled as a sample. A scoopful of each leftover feed sample (n = 17) approximately 100 g was collected and placed in a disposable bag. Live houseflies (*Musca domestica*) (n = 14) were trapped by using adhesive fly trap placed at two spots in the farm. By using some sterile forceps, ten live houseflies were taken from the adhesive flytrap and put into a transport media and was considered as a pooled sample. Two pooled samples of houseflies were collected from each of the seven farms, and one pooled sample each for the remaining three farms. Drinking water (n = 16) were taken directly from the dairy cattle water trough, while sources of drinking water (n = 10) were taken if the sources were untreated pond or well. At least 100 mL of drinking water and source of drinking water were each collected in an individual sterile bottle. All swab samples were kept in BPW.

### 4.2. Isolation and Identification of ESBL-Producing E. coli

The isolation and detection of ESBL-producing *E. coli* involved two stages. The first stage involved isolation and identification of *E. coli* in all samples collected, and phenotypic detection of ESBL *E. coli* in the second stage. Using a sterile streaking loop, each sample of faecal swabs, floors, and feed and water troughs was cultured on Chromocult^®^ Coliform Agar (Merck KGaA, Darmstadt, Germany) (a selective, chromogenic media). Each feed sample was mixed with 250 mL BPW and was left for 30 m at room temperature to soak. By using a sterile pipette, 0.5 mL of the sample mixture was cultured on the agar. Each pooled sample of flies were crushed with a sterile swab. Using a sterile streaking loop, loopfuls of each sample were streaked on the agar. Each drinking water and source of drinking water sample was poured into a sterile water filter apparatus; a sterile membrane filter with pore size 0.45 mm was used. The membrane filter was then removed and placed onto the agar. Following incubation, 0.5 mL of the milk sample was inoculated on the agar. All the inoculated agar plates were incubated at 37 °C for 18–24 h. The dark-blue to violet colonies, which appeared on Chromocult^®^ Coliform Agar, were presumptively identified as *E. coli*. These colonies were overlaid with a drop of Kovacs^®^ Indole reagent. The presence of *E. coli* is positive and confirmed if a cherry-red colour appeared after a few seconds. All isolates were then confirmed using PCR assay. Positive *E. coli* colonies isolated from faeces, the farm environment, and milk samples were further cultured on CHROMagar™ ESBL (CHROMagar™, Paris, France) and incubated at 37 °C for 18 to 24 h. This media is a selective and chromogenic media for isolation of ESBL *E. coli*. Suspected ESBL-producing *E. coli* colonies were subcultured on Nutrient agar (CM0003, Oxoid Ltd., Basingstoke, Hampshire, England) prior to the phenotypic confirmatory tests.

### 4.3. Phenotypic Confirmation of ESBL-Producing E. coli

Confirmation of ESBL *E. coli* was carried out using the double disk diffusion method [34]. All presumptive ESBL *E. coli* isolates were subjected to confirmation for ESBL production using the combination disk diffusion method with ceftazidime (30 µg) and cefotaxime (30 µg) alone and in combination with clavulanic acid (30 µg/10 µg) (Difco/BD, Franklin Lakes, NJ, USA). The *E. coli* isolates were phenotypically considered as ESBL-producer, when an increase in the size of inhibition zone is greater than (≥5 mm) for antimicrobial agent with or without clavulanic acid was observed. *Escherichia coli* ATCC 25922, *Staphylococcus aureus* ATCC 29213, and *Pseudomonas aeruginosa* ATCC 27853 were used as quality control strains.

### 4.4. Genomic DNA Extraction

Genomic DNA extraction was performed using boiling method as described by Li et al. [34] with slight modification (i.e., bacterial cells were heat treated at 98 °C instead of 100 °C). Suspension of overnight fresh cultures of ESBL *E. coli* isolates was prepared using a sterile distilled water in a 100 µL micro-centrifuge tubes (Eppendorf). Cell suspensions were heated using a dry bath at 98 °C for 10 m followed by cooling at room temperature for 5–10 m prior to centrifuging at 13,000× *g* for 3 m at 25 °C. Total extracted DNA (200 ng equivalent to 5 µL measured with the aid of a spectrophometer) was then subjected to multiplex polymerase chain reaction (mPCR) assay.

### 4.5. Genotypic Detection of ESBL Genes by mPCR

All the 18 ESBL *E. coli* isolates were investigated for the presence of ESBL genotypes, including TEM (643 bp), SHV (168 bp), CTX-M (402 bp), and OXA (250 bp) by mPCR assay. A list of primers used for mPCR assay is shown in Table 4. The mPCR assay was performed in a 50 µL reaction mixture containing 10 µM of primers set (1 µL each primer), 25 µL (5U/µL) MyTaq™ HS Mix (Bioline, London, UK), and 10 µL RNAse free water (Qiagen, Hilden, Germany). The multiplex PCR cycling condition was as follows: initial denaturation at 95 °C for 1 m followed by 30 cycles of denaturation at 95 °C for 15 s, annealing at 60 °C for 15 s, extension at 72 °C for 10 s, and final extension at 72 °C for 10 m [14]. Amplified mPCR products were resolved in 1.0% agarose gel containing ethidium bromide. Quality control organisms used in this study were *K. pneumoniae* ATCC 700603 as positive control and *E. coli* ATCC 25922 as negative control [35].

### 4.6. Data Analysis

Data were analysed using IBM SPSS Statistics version 21.0. The chi-square (χ^2^) test was used to compare the occurrence of ESBL-producing *E. coli* in dairy cattle, the farm environment, and milk samples. Statistical significance was defined at 95% confidence interval (*p* ≤ 0.05).

## 5. Conclusions

In conclusion, this study revealed the occurrence of a slightly significant proportion of ESBL-producing *E. coli* in dairy cattle, the farm environment, and milk. It also represents the occurrence of CTX-M enzyme as being the predominant ESBL genotype from cattle. Moreover, it indicated that cattle, milk, and the farm environment can serve as potential reservoirs of ESBL genes that may perpetuate the spread and maintenance of ESBL genes in human, animals, and the environment.

## Figures and Tables

**Table 1:** Occurrence of ESBL-producing *E. coli* in dairy cattle, farm environment, and milk.

Farms	No. of Samples Collected	Sample Type			No. of Positive ESBL-Producing *E*. *coli* (%)
		Faecal Samples *n* = 229	Farm Environment *n* = 77	Milk *n* = 71	
1	38	0	0	1	1 (2.6)
2	42	0	1	-	1 (2.4)
3	44	0	2	1	3 (6.8)
4	44	1	0	6	7 (15.9)
5	26	0	1	1	2 (7.7)
6	44	0	0	0	0 (0)
7	28	0	1	0	1 (3.6)
8	35	0	0	0	0 (0)
9	39	0	0	3	3 (7.7)
10	37	0	0	0	0 (0)
TOTAL	377	1 (0.27%)	5 (1.32%)	12 (3.18%)	18 (4.8)

**Table 2:** Occurrence of ESBL-producing *E. coli* in farm environment.

Sample Type	No. of Samples Collected	No. of Positive ESBL-Producing *E. coli* (%)
Floor, feed, and water trough swabs	20	0 (0)
Drinking water	16	3 (18.6)
Source of drinking water	10	1 (10)
Feed	17	0 (0)
House flies (*Musca domestica*)	14	1 (7.1)
TOTAL	77	5 (6.5)

**Table 3:** ESBL genotypes detected in ESBL-producing *E. coli*.

Farms	Sample ID (18 Isolates)	Sample Type	ESBL Genotype	Genetic Profile
1	F1M3	Milk	TEM	I
2	F2WS	Source of drinking water	CTX-M	II
3	F2M8	Milk	CTX-M	II
	F3DW1	Drinking water	TEM, CTX-M	III
	F3DW2	Drinking water	CTX-M	II
4	F4M1	Milk	Not detected	IV
	F4M3	Milk	TEM, CTX-M	III
	F4M4	Milk	TEM, CTX-M	III
	F4M5	Milk	TEM, CTX-M	III
	F4M6	Milk	TEM, CTX-M	III
	F4M7	Milk	CTX-M	II
	F4M4	Faeces	TEM, CTX-M	III
5	F5M4	Milk	Not detected	IV
	F5Hf 2	House flies	Not detected	IV
7	F7DW2	Drinking water	TEM, CTX-M	III
9	F7M5	Milk	TEM, CTX-M	III
	F9M7	Milk	Not detected	IV
	F9M8	Milk	TEM	I

F1, F2, F3, F4, F5, F7, F9 (represents farms visited) M1, M3, M4, M5, M6, M7, M8, WS, DW1, DW2, Hf2 (represents number and type of samples from each farms), I, II, III, IV (represents genetic profiles of the isolates).

**Table 4:** Primers used for the detection of ESBL genes and *E. coli*.

Gene	Primer Sequence (5′–3′ Direction)	Product Size (bp)	Gene Accession No.
TEM	Forward—TCCTTGAGAGTTTTCGCCCC Reverse—TGACTCCCCGTCGTGTAGAT	643	EU352903
SHV	Forward—CAATCACGACGGCGGAATCT Reverse—GTGGGTCATGTCGGTACCAT	168	AB731686
CTX-M	Forward—AAGCACGTCAATGGGACGAT Reverse—GTTGGTGGTGCCATAGCCA	402	JN411912
OXA	Forward—TTGCACTTGATAGTGGTGTGA Reverse—AGTGAGTTGTCAAGCCAAAAAGT	250	JN003412
*E. coli*	Forward—TGACGTTACCCGCAGAAGAA Reverse—CTCCAATCCGGACTACGACG	832	X80724
